# Selection

**Published:** 1902-03

**Authors:** 


### SELECTION.

### UNITED STATES DEPARTMENT OF AGRICULTURE, DIVISION OF PUBLICATIONS—JANUARY BULLETIN.

### The Improved Respiration Calorimeter for Studying the Nutrition of Man.

About five years ago the public interest was excited in the nutrition investigations carried on as a co-operative enterprise by the United States Department of Agriculture, the Storrs Experiment Station, and Wesleyan University, at Middletown, Conn., by the experiments on the nutrition of men in the respiration calorimeter. The "man in the box" was overwhelmed with visitors curious to see a man housed in an air-tight copper box for from four to twelve days. Each year the experts in charge of the apparatus have made changes tending to simplify its manipulation and increase its accuracy. The original copper box is no more. For the past three months a force of mechanicians and electricians have been at work entirely reconstructing this remarkable apparatus, and it is expected that the finishing touches will be given in a few days. It is believed that the new apparatus will be very accurate. In the original box the heat-measuring devices were so delicate as to permit of the measurement of the heat developed in so slight a motion as rising from a chair, but it is hoped that the new apparatus will record the heat given off in a sudden cough or sneeze or change of position in bed.

The new devices include an unusually accurate thermometer for recording changes of body temperature, an improved cooling apparatus to remove the heat radiated from the body, and a novel method of introducing food and drink into the air-tight chamber without admitting air. This latter device is on the principle of the double-door chamber used in tunnel construction. The chamber between the doors is used for the transitory storage of material, only one door being open at a time.

The double insulated wooden walls have been coated inside and outside with asbestos paper, to prevent any possible danger from fire due to breaks in the numerous electric circuits. The chamber is literally inclosed in a web or network of wires, each with its own independent connection, and all leading to a table outside, where an observer sits continually, day in and day out. An especially elaborate and ingenious switch of a new type, controlling all the electric circuits, is now completed, ready for installation. The object is to regulate the temperature of the interior of the chamber and measure the heat given off from the body of the occupant.

The system of plumbing is hardly less intricate than that of electric wiring, for each wire has its counterpart in a water pipe. The pipes are used to cool the air spaces around the metal box, and the wires serve as a resistance to the electric currents, and thereby heat the air correspondingly.

The box rests on lignum vitæ castors in a track, and when rolled into its casing, or shed, the double back is put in place and the metal box is completely housed. A telephone system permits conversation with the outside world, and aside from what he hears over the telephone the subject is dead to the sound of voices when in the chamber. A large water meter measures the amount of water used to cool the inside of the insulated chamber and bring away the heat generated by the body. Long thermometers, graduated to fiftieths of a degree, record the temperature of the cold water as it enters the heat-absorbing system and the warmed water as it comes out. A new mechanical pump provides for a very constant ventilation. The large double-glass window offers a good opportunity to see the subject inside, and it is seldom that his every motion is not watched either by some assistant or by a visitor to the university laboratory.

During certain kinds of experiments the subject must engage in hard muscular work. The small size of the innermost chamber forbids even ordinary gymnastics or calisthenics, and the work must be done on a machine. The new work machine consists of a stationary bicycle so arranged that its rear tire is brought into contact with a pulley or a small electric dynamo, the pulley being grooved to fit the curve of the rubber tire. The machine is geared to 96, and when riding at the fastest pace the armature of the dynamo runs at 3500 revolutions per minute. The dynamo is magnetized by a current from outside, and the amount of electricity generated by the man pedaling the bicycle is carefully measured. He works eight hours a day, in four periods of two hours each. The equivalent distance ridden on a smooth track would be nearly one hundred miles—a good day's work.

During the years in which the apparatus has been used a large amount of experimental results has accumulated. The work done is already more extensive, more accurate, and more complete than found in any other inquiry. A bulletin of the Office of Experiment Stations will soon be issued, giving the results of a large number of experiments. Another bulletin is now in preparation, which will give still more experiments and summaries of results up to the present date. They throw most valuable light upon the changes of matter and energy which take place as the food is used in the body. The experiments number forty-eight, and were made with five different men. They cover altogether 155 experimental days and nights. The men have had different kinds and amounts of food, and the tests have been made when they were at rest and when they were engaged in more or less severe muscular work. The results are very important in their bearing upon the theory of nutrition and the practical uses and values of food.

